# Effect of normobaric hypoxic exercise on blood pressure in old individuals

**DOI:** 10.1007/s00421-020-04572-6

**Published:** 2020-12-23

**Authors:** Markus Hein, Kristine Chobanyan-Jürgens, Uwe Tegtbur, Stefan Engeli, Jens Jordan, Sven Haufe

**Affiliations:** 1 Institute of Sports Medicine, Hannover Medical School, Carl-Neuberg-Str. 1, 30625 Hannover, Germany; 2 Institute of Clinical Pharmacology, Hannover Medical School, Hannover, Germany; 3 Department of Clinical Pharmacology and Pharmacoepidemiology, Heidelberg University Hospital, Heidelberg, Germany; 4 Institute of Aerospace Medicine, German Aerospace Center and University of Cologne, Cologne, Germany

**Keywords:** Hypoxia, Exercise, Blood pressure, Elderly

## Abstract

**Purpose:**

To test the hypothesis that the combination of endurance training and hypoxia leads to greater improvements in resting and exercise blood pressure in old sedentary individuals compared to endurance training only.

**Methods:**

We randomly assigned 29 old overweight participants (age: 62 ± 6 years, body mass index (BMI): 28.5 ± 0.5 kg/m^2^, 52% men) to single blind 8-week bicycle exercise in hypoxia (fraction of inspired oxygen (F_I_O_2_) = 0.15) or normoxia (F_I_O_2_ = 0.21). Brachial blood pressure was measured at rest, during maximal incremental exercise testing, and during a 30 min constant work rate test, at baseline and after the training period.

**Results:**

Work rate, heart rate and perceived exertion during training were similar in both groups, with lower oxygen saturation for participants exercising under hypoxia (88.7 ± 1.5 vs. 96.2 ± 1.2%, *t*(27) = − 13.04, *p* < 0.001, |*g*|= 4.85). Office blood pressure and blood pressure during incremental exercise tests did not change significantly in either group after the training program. Systolic blood pressure during the constant work rate test was reduced after training in hypoxia (160 ± 18 vs. 151 ± 14 mmHg, *t*(13) = 2.44 *p* < 0.05, |*d*|= 0.55) but not normoxia (154 ± 22 vs. 150 ± 16 mmHg, *t*(14) = 0.75, *p* = 0.46, |*d*|= 0.18) with no difference between groups over time (*F* = 0.08, *p* = 0.77, *η*^2^ = 0.01).

**Conclusion:**

In old individuals hypoxia in addition to exercise does not have superior effects on office or exercise blood pressure compared to training in normoxia.

**Trial registration number:**

ClinicalTrials.gov No. NCT02196623 (registered 22 July 2014).

## Introduction

Hypertension is a major risk factor for death (Whelton et al. 2017) and predicts disability-adjusted life years (Lim et al. 2012; Whelton et al. 2017). While aerobic capacity decreases, systolic blood pressure progressively increases (Benetos et al. 2002) with advancing age. Moreover, in old individuals, elevated blood pressure is associated with substantially higher absolute cardiovascular risk than in young individuals (Rapsomaniki et al. 2014).

Physical activity improves blood pressure control (Cornelissen and Fagard 2005) in patients with arterial hypertension (Somers et al. 1991) and is equally effective in lowering systolic blood pressure as commonly prescribed antihypertensive medications (e.g. angiotensin-converting enzyme inhibitors, angiotensin-2 receptor antagonists, β-adrenoceptor antagonists, calcium channel blockers and diuretics) (Naci et al. 2019). However, endurance training may be limited by orthopedic comorbidities in old individuals. Training strategies that reduce the strain on the locomotor system, such as hypoxic exercise, may be of interest to practitioners working with this population. Thereby, less stress might be imposed on the musculoskeletal system while still realizing the same or even greater cardiovascular benefits of exercising (Haufe et al. 2008; Pramsohler et al. 2017).

The current data concerning the blood pressure response to hypoxia are contradictory. Intermittent hypoxia comprising intervals of breathing normoxic and hypoxic air without physical exercise has been previously tested in treating patients with arterial hypertension (Serebrovskaya et al. 2008). On the other hand, hypoxia may play a role in the pathogenesis of arterial hypertension (Kayser and Verges 2013) through repeated fluctuations in intrathoracic pressure caused by increased activity of the chemoreceptor-mediated sympathetic nervous system, resulting in increased heart rate, cardiac output and peripheral resistance (Malfatto, Ochoa and Parati 2009).

Breathing hypoxic air engages similar metabolic pathways as endurance exercise such as the hypoxia inducible factor 1 system (Ameln et al. 2005). Up regulation of hypoxia inducible factor 1α (HIF-1α) protein expression during exercise could further enhance activation of vascular endothelial growth factor (VEGF) (Ameln et al. 2005). VEGF plays a critical role in blood pressure control via nitric oxide synthase expression and nitric oxide activity (Facemire et al. 2009). Hypoxia-induced VEGF activation may also contribute to increased capillarization, which is also a typical response to endurance exercise (Hansen et al. 2010).

Previous studies have evaluated the combination of exercise in hypoxia for several health-related outcomes (González-Muniesa et al. 2015), yet, data from randomized controlled trials on blood pressure changes following hypoxic exercise are sparse. We tested the hypothesis that the combination of exercise and hypoxia lead to greater improvements in resting and exercise blood pressure compared to normoxic endurance exercise in old sedentary individuals.

## Methods

This is a secondary analysis of a study testing the effects of hypoxic training on changes in whole body insulin sensitivity and oxidative metabolism as primary outcome (Chobanyan-Jürgens et al. 2019) (ClinicalTrials.gov Identifier: NCT02196623). We included women and men aged 55–75 years who were weight stable during the last 6 months (± 2% body weight), had a body mass index between 20 and 35 kg/m^2^, and a homeostasis model assessment insulin resistance index (HOMA-IR) at screening between 2.0 and 4.0 units. Sixty-eight individuals were screened for the study of which 29 met the inclusion criteria and were randomized to hypoxic or normoxic training.

Detailed medical history, examination, 12-lead electrocardiogram, and blood sampling for routine laboratory tests assessed preexisting diseases. Exclusion criteria were more than 1 h of scheduled exercise training per week; known diagnosis of type 2 diabetes or HbA1c > 6.5% (> 48 mmol/mol); smoking > 20 cigarette/day; known alcohol or drug abuse; acute or chronic infections; increased bleeding risk by history or laboratory testing; any contraindication (orthopedic, cardiopulmonary etc.) to perform exercise training. The institutional review board of Hannover Medical School approved the study, and we obtained written informed consent before participants’ enrollment.

The training program was reported in detail previously (Chobanyan-Jürgens et al. 2019). In brief, participants completed an 8-week supervised endurance training on a stationary bicycle ergometer (Optibike 50, ergoline, Bitz, Germany) in a normobaric hypoxic chamber, with ambient conditions (hypoxic [F_I_O_2_ = 0.15, corresponding to 2750 m altitude] or normoxic [F_I_O_2_ = 0.21]) in a single-blinded fashion. Participants trained thrice weekly at similar relative exercise intensities in both intervention groups (heart rate corresponding to 60% of pre-training peak oxygen consumption (*V*O_2peak_)) for 30 min. To compensate for training-induced effects we increased exercise intensity to 70% of pre-training *V*O_2peak_ and exercise duration to 40 min after the first 4 weeks). Heart rate and blood oxygen saturation (Rad-5, Masimo Corporation, Irvine, USA) were measured every ten minutes during training and rating of perceived exertion (RPE) (Borg scale) was documented at the end of every session.

Before (baseline) and after the training program (post), participants reported to the laboratory twice. On the first day participants rested on a chair in a quiet room for five minutes, before office blood pressure and heart rate (CARESCAPE V100, GE Healthcare, Boston, USA) were measured three times in one-minute intervals. Results are given as average of the three measurements. Afterwards, anthropometric data and body composition (BodPod, Life Measurement, Inc., Concord, USA) were determined. Finally participants performed a graded exercise test on a bicycle ergometer (ergometrics 900, ergoline, Bitz, Germany) starting at a work rate of 50 W increasing by 10 W every minute until volitional exhaustion. Throughout the test, we recorded heart rate using a 12-channel-electrocardiogram (CardioSoft, GE Healthcare, Boston, USA) and respiratory gas exchange breath-by-breath using indirect calorimetry (Masterscreen CPX, Becton Dickinson, Franklin Lakes, USA). Every three minutes, we measured blood pressure and obtained small blood samples from the hyperaemized earlobe to measure blood lactate concentrations (Biosen S-Line, EKF-diagnostic GmbH, Barleben, Germany). We determined *V*O_2peak_ and maximal heart rate as the highest 30 s average during the exercise test.

With at least two days rest in between, participants performed a submaximal exercise test at a work rate corresponding to 60% of their baseline *V*O_2peak_ for 30 min. We recorded heart rate and respiratory gas exchange as described above and blood pressure and blood lactate concentrations every five minutes throughout the test. Average heart rate, blood lactate concentration and respiratory exchange ratio were calculated as mean ± standard deviation (SD) from minute 5 to 30. At the end of the test participants reported their RPE. After the training program, the submaximal test was performed at the same absolute work rate as at baseline and at least two days before the graded exercise test.

### Statistical analysis

We conducted the statistical analysis in the intention-to-treat population, defined as all randomized individuals who participated in at least one training session. Missing values were replaced by baseline values. As sensitivity analysis, we also performed a per-protocol analysis (only participants with pre- and post-intervention data and at least 23 out of 24 scheduled training sessions). Differences between groups at baseline were analyzed with an unpaired student’s *t* test. Within-group differences in blood pressures changes from pre- to post-training for the intervention and control group were calculated with student’s t test for paired samples. Mean differences between groups over time were analyzed with an analysis of covariance (ANCOVA), adjusted for sex, age and the baseline value as covariates. Effect sizes for differences between groups were calculated using Hedges’g (|*g*|) and for differences within groups (baseline–post) with Cohen’s *d* (|*d*|). For the ANCOVA, Eta-squared (*η*^2^) was used to calculate effect sizes between groups over time. Univariate associations between parameters were tested using Spearman’s correlation coefficient.

Values are presented as mean ± standard deviation (SD) unless otherwise stated. *p* < 0.05 was considered to be statistically significant. All analyses were carried out with the SPSS software package for Windows^®^ (Version 24, IBM Corp., Armonk, NY, USA).

## Results

Twelve participants in the hypoxia group and thirteen participants in the normoxia group completed the training protocol. Two participants dropped out prior to completing the post-training tests in each group due to study unrelated events. For all outcomes assessed no further values had to be replaced post intervention, except for one individual in the normoxia group who did not perform the constant work rate test after the intervention and missing values were therefore replaced by the respective baseline values.

Demographic and physiological baseline characteristics for both treatment groups are given in Table 1. Average training work rate was 72 ± 23 W in the hypoxia group and 83 ± 33 W in the normoxia group (*t*(27) = − 1.00, *p* = 0.33, |*g*|= 0.37). Both groups trained at similar absolute heart rates (hypoxia: 114 ± 14 bpm, normoxia: 115 ± 10 bpm, (*t*(27) = − 0.89, *p* = 0.91, |*g*|= 0.04) and similar percentage of their maximal heart rate (hypoxia: 76 ± 6%, normoxia: 74 ± 5% (*t*(27) = − 0.12, *p* = 0.30, |*g*|= 0.40). Arterial oxygen saturation during training was significantly lower in the hypoxia (89 ± 1.5%) compared to the normoxia group (96 ± 1.5%) (*t*(27) = − 13.04, *p* < 0.001, |*g*|= 4.85). Exercise performance parameters for the initial session of each week (training progression) are given in Table 2. Of those participants completing the 8-week intervention (*n* = 25) seventeen performed 24 out of 24 scheduled exercise training sessions (68%) and five performed 23 out of 24 scheduled sessions (24%). One individual in the normoxia and one in the hypoxia group performed 15 out of 24 exercise sessions (8%).

**Table 1:** Demographic and physiological baseline characteristics

	Hypoxia group	Normoxia group	*p*	|*g*|
Age, years	60.4 ± 5.0	63.8 ± 5.8	0.10	0.63
Body weight, kg	86.7 ± 10.3	88.4 ± 14.5	0.72	0.13
BMI, kg*m^−2^	28.6 ± 3.0	28.4 ± 1.9	0.71	0.14
RR sys, mmHg	129.1 ± 9.3	133.9 ± 13.5	0.27	0.42
RR dia, mmHg	81.6 ± 8.5	79.2 ± 10.3	0.49	0.26
Resting heart rate, bpm	68 ± 7	66 ± 9	0.63	0.18

Data are mean ± SD. No significant differences between groups as analyzed with Student’s *t* test for unpaired samples. |*g*|: Hedges’*g*

*BMI* body mass index, *RR* sys systolic blood pressure, *RR* dia diastolic blood pressure

**Table 2:** Variables during training for the first session of any week

Week	work rate (watt/kg)				heart rate (bpm)				RPE (Borg Scale)				SaO_2_ (%)			
	Hypoxia	Normoxia	*p* value	|*g*|	Hypoxia	Normoxia	*p* value	|*g*|	Hypoxia	Normoxia	*p* value	|*g*|	Hypoxia	Normoxia	*p* value	|*g*|
1	0.77 ± 0.24	0.84 ± 0.27	0.48	0.27	112 ± 16	113 ± 11	0.83	0.05	13 ± 1.1	12 ± 0.7	0.36	0.35	89.0 ± 2.1	96.4 ± 1.1	< 0.01	4.31
2	0.77 ± 0.24	0.85 ± 0.27	0.39	0.33	112 ± 14	112 ± 12	0.95	0.01	13 ± 1.2	12 ± 0.9	0.88	0.06	88.9 ± 1.6	96.3 ± 1.2	< 0.01	5.27
3	0.79 ± 0.26	0.87 ± 0.28	0.45	0.30	113 ± 14	113 ± 9	0.95	0.01	12 ± 0.9	13 ± 0.9	0.81	0.09	88.6 ± 2.1	96.4 ± 1.4	< 0.01	4.44
4	0.80 ± 0.25	0.87 ± 0.28	0.46	0.29	113 ± 16	113 ± 9	0.99	0.00	13 ± 1.2	12 ± 0.8	0.76	0.12	88.9 ± 2.3	96.2 ± 1.3	< 0.01	4.00
5	0.87 ± 0.28	0.96 ± 0.29	0.46	0.29	116 ± 15	117 ± 11	0.88	0.03	14 ± 1.0	13 ± 0.9	0.42	0.32	88.8 ± 1.6	96.1 ± 1.2	< 0.01	5.20
6	0.90 ± 0.28	0.95 ± 0.30	0.70	0.29	116 ± 13	119 ± 12	0.19	0.28	14 ± 1.3	13 ± 1.2	0.48	0.30	88.3 ± 1.8	96.2 ± 1.5	< 0.01	4.83
7	0.91 ± 0.26	0.94 ± 0.29	0.78	0.21	114 ± 14	118 ± 11	0.19	0.28	13 ± 1.3	13 ± 1.0	0.35	0.40	88.0 ± 1.7	96.2 ± 1.4	< 0.01	5.26
8	0.93 ± 0.28	0.94 ± 0.29	0.88	0.06	116 ± 14	116 ± 11	0.98	0.00	13 ± 1.6	13 ± 1.3	0.81	0.10	88.8 ± 2.2	96.2 ± 1.5	< 0.01	4.01

Data are mean ± SD, mean heart rate and SaO_2_ was calculated from minute 10–30 for week 1–5 and from minute 10–40 from week 5–8. |*g*|: Hedges´*g*

*RPE* rating of perceived exertion, *SaO*_*2*_ oxygen saturation

After the training program body weight and body fat mass (hypoxia group: − 0.5 ± 2.3 kg; normoxia group: –0.7 ± 1.6 kg; between-groups: *t*(27) = 0.26, *p* = 0.80, |*g*|= 0.10) did not change significantly with training irrespective of oxygen conditions. The improvement of *V*O_2peak_ was only statistically significant in the normoxia training group (Table 3) with no differences between groups.

**Table 3:** Performance variables during graded exercise test

	Hypoxia group			Normoxia group			Group x time	
	Baseline	Post	|*d*|	Baseline	Post	|*d*|	*p* value	*η*^2^
Maximal power, W	154 ± 41	167 ± 46*	0.30	161 ± 55	169 ± 56*	0.14	0.29	0.05
Relative maximal power, W/kg	1.8 ± 0.4	2.0 ± 0.5*	0.44	1.8 ± 0.4	1.9 ± 0.5*	0.22	0.13	0.09
VO_2_peak, ml/min	2090 ± 501	2232 ± 587	0.26	2090 ± 665	2210 ± 636*	0.18	0.60	0.01

Data are mean ± SD, * *p* < 0.05

|*d*| Cohen´s *d*, *η*^2^ eta-squared, *VO*_*2*_ oxygen uptake

**Fig. 1:**
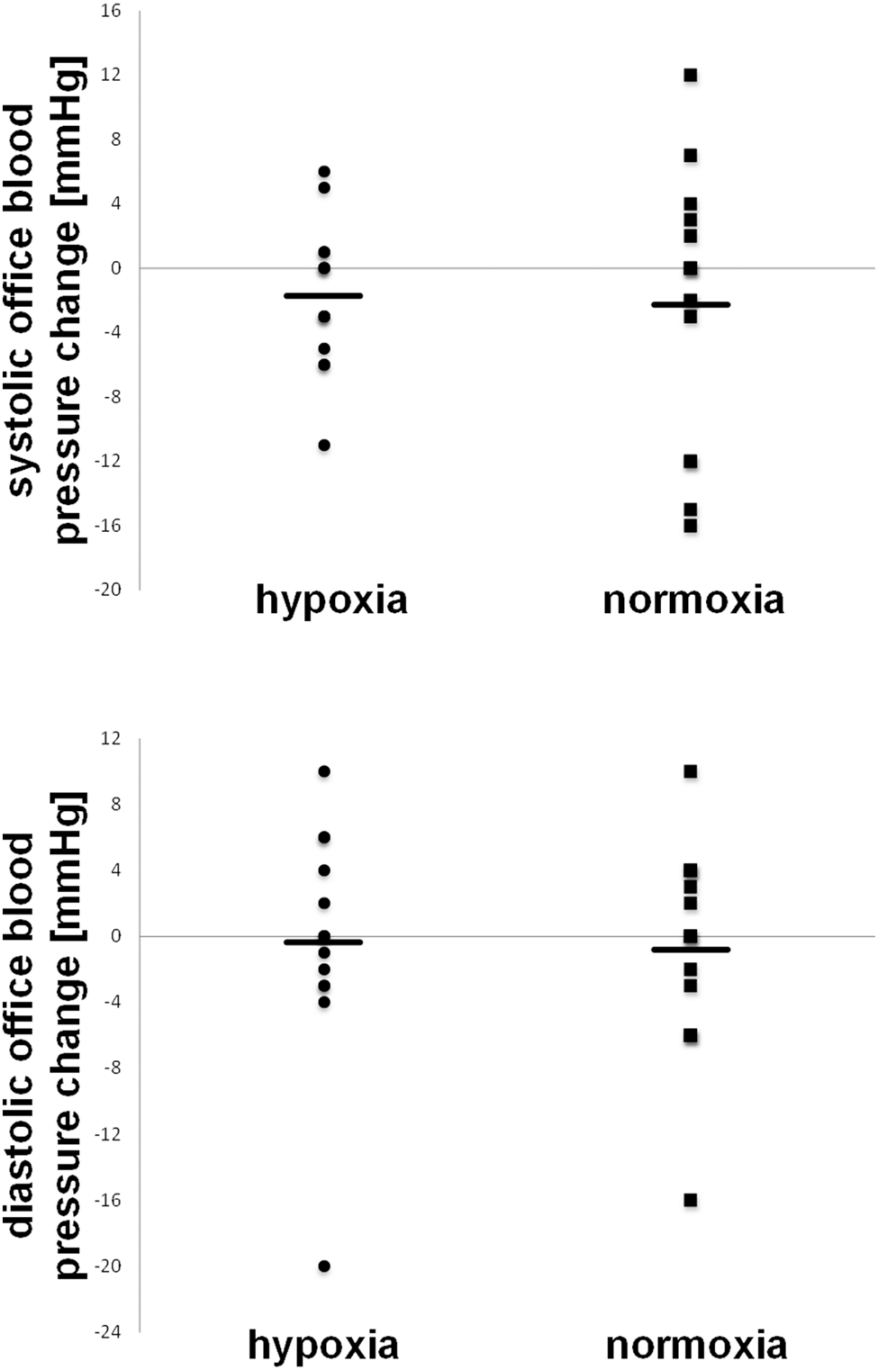
Individual and mean (solid line) changes in office blood pressure of participants randomized to exercise in normoxia or hypoxia after 8 weeks of training compared to baseline. No significant differences were detected within groups or between groups over time

Eight weeks of endurance training in hypoxic and normoxic conditions did not significantly change systolic (hypoxia baseline: 129 ± 9 mmHg; post: 127 ± 8 mmHg, *t*(13) = 1.42, *p* = 0.18, |*d*|= 0.21; normoxia baseline: 134 ± 14 mmHg; post: 132 ± 12 mmHg, *t*(14) = 1.08 *p* = 0.30, |*d*|= 0.17) or diastolic (hypoxia baseline: 82 ± 9 mmHg; post: 81 ± 7 mmHg, *t*(13) = 0.19, *p* = 0.85, |*d*|= 0.05; normoxia baseline: 79 ± 10 mmHg; post: 78 ± 11 mmHg, *t*(14) = 0.50 *p* = 0.62, |*d*|= 0.08) office blood pressure (see also Fig. 1). A per-protocol analysis (*n* = 23) showed similar results for office systolic (hypoxia baseline: 128 ± 9 mmHg; post: 126 ± 8 mmHg, *t*(10) = 1.12, *p* = 0.29, |*d*|= 0.20; normoxia baseline: 134 ± 12 mmHg; post: 132 ± 11 mmHg, *t*(11) = 0.73 *p* = 0.48, |*d*|= 0.16) and diastolic blood pressure (hypoxia baseline: 81 ± 9 mmHg; post: 81 ± 7 mmHg, *t*(10) = 0.69, *p* = 0.35, |*d*|= 0.18; normoxia baseline: 81 ± 9 mmHg; post: 80 ± 10 mmHg, *t*(11) = 0.26 *p* = 0.80, |*d*|= 0.05).

**Fig. 2:**
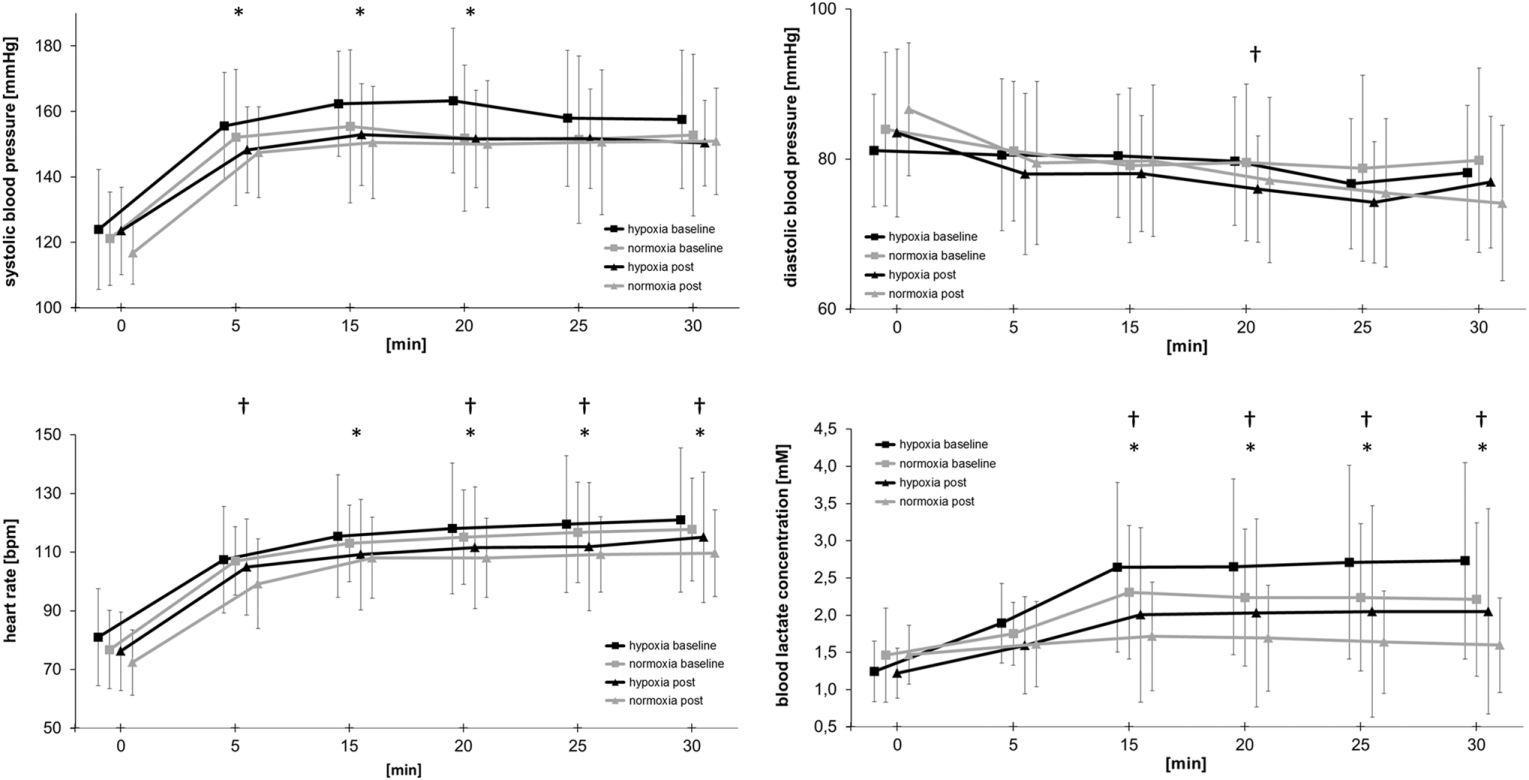
Blood pressure, heart rate, and blood lactate concentration during constant work rate exercise test (60% of baseline peak oxygen uptake) on a bicycle ergometer at baseline and after 8 weeks. Data are mean ± SD. *p < 0.05 between baseline and post in the hypoxia group. **†p** < 0.05 between baseline and post in the normoxia group. No significant differences were detected between groups over time

During the constant work rate exercise test, systolic blood pressure was significantly reduced after training within the hypoxia group, (160 ± 18 to 151 ± 14 mmHg, *t*(13) = 2.44 *p* < 0.05, |*d*|= 0.55), but not the normoxia group (154 ± 22 mmHg to 150 ± 16 mmHg, *t*(14) = 0.75, *p* = 0.46, |*d*|= 0.18), (see also Fig. 2), with no differences between groups over time (*F* = 0.08, *p* = 0.77, *η*^2^ = 0.01). Both groups showed reduced average heart rate (hypoxia baseline: 116 ± 21 bpm; post: 110 ± 20 bpm, *t*(13) = 2.39, *p* < 0.05, |*d*|= 0.29; normoxia baseline: 114 ± 14 bpm; post: 107 ± 13 bpm, *t*(14) = 2.40 *p* < 0.05, |*d*|= 0.48), blood lactate concentration (hypoxia baseline: 2.5 ± 1.1 mM; post: 1.9 ± 1.2 mM, *t*(13) = 3.48, *p* < 0.05, |*d*|= 0.52; normoxia baseline: 2.2 ± 0.8 mM; post: 1.7 ± 0.7 mM, *t*(14) = 2.97 *p* < 0.05, |*d*|= 0.65) (see also Fig. 2 for data at single time points), respiratory exchange ratio (hypoxia baseline: 0.97 ± 0.05; post: 0.94 ± 0.06, *t*(13) = 2.48, *p* < 0.05, |*d*|= 0.59; normoxia baseline: 0.95 ± 0.04; post: 0.92 ± 0.04, *t*(14) = 2.18 *p* < 0.05, |*d*|= 0.63) and perceived exertion (hypoxia baseline: 13.5 ± 1.3; post: 12.8 ± 1.6, *t*(13) = 2.68, *p* < 0.05, |*d*|= 0.48; normoxia baseline: 12.8 ± 0.7; post: 11.7 ± 1.8, *t*(14) = 2.87 *p* < 0.05, |*d*|= 0.79) during constant work rate tests after the training program.

We did not observe significant correlations between training compliance (percentage of completed exercise sessions) and intervention-induced changes in blood pressure at rest (systolic BP: Rho: 0.01, *p* = 0.98; diastolic BP: Rho: 0.08, p = 0.67) or during constant submaximal exercise (systolic BP: Rho: 0.17, *p* = 0.38; diastolic BP: Rho: 0.07, *p* = 0.72).

## Discussion

The main finding of our study is that 8 weeks hypoxic endurance training had no major and consistent effect beyond that of exercise in normoxia on resting and exercise blood pressure in old sedentary individuals.

Age-induced blood pressure elevation is thought to be mainly due to atherosclerotic changes, stiffening of large arteries, renal dysfunction, and arterial baroreflex impairment (Benetos et al. 2002). Many of the changes occurring with advanced age are associated with a decrease in physical activity and cardiorespiratory fitness (Milanović et al. 2013). Physical training can be a potential treatment strategy to reduce blood pressure, but its effects are controversial and may vary between individuals (Cornelissen and Fagard 2005; Somers et al. 1991).

Beneficial effects of regular hypoxic training on blood pressure regulation have been observed in some studies. Three weeks of moderate sports activities at natural 1700 m altitude showed a significant decrease in systolic and diastolic blood pressure in individuals with metabolic syndrome (Schobersberger et al. 2003). Kong et al. and Morishima et al. reported a 10 mmHg and 7 mmHg decrease in systolic blood pressure after 4 weeks exercise training at 16.4–14.5% F_I_O_2_ (Kong et al. 2014; Morishima et al. 2015), whereas González-Muniesa et al. only observed a reduction in diastolic blood pressure after an 8-week strength and endurance protocol in normobaric hypoxia simulating 2000–3350 m altitude (González-Muniesa et al. 2015). We could not confirm these results in our cohort. Most studies reported improved body composition after the training period which is a crucial factor in decreasing blood pressure by exercise (Stewart et al. 2005). In our study individuals´ weight remained stable, which might partly explain different outcomes to other studies in which weight loss might also be a result of the additional dietary advice given to participants (González-Muniesa et al. 2015; Kong et al. 2014; Schobersberger et al. 2003). Further reasons for the lack of favorable effects of our tested hypoxic training program might include insufficient duration, frequency and severity of the hypoxic episodes as these seems to be critical factors in determining whether intermittent hypoxia is beneficial (Manukhina et al. 2006; Serebrovskaya and Xi 2016). Moreover, there is some evidence that old individuals might be resistant to exercise-induced blood pressure reduction (Stewart et al. 2005) and the change of body composition is a decisive factor in this regard (Kong et al. 2014; Stewart et al. 2005).

Yet, our results are in line with some other studies where hypoxic exercise did not have significant positive effects on arterial blood pressure Fu et al. 2007: (systolic: ± 0 mmHg, diastolic: + 1 mmHg), Gatterer et al. 2015 (systolic: − 4 mmHg, diastolic: − 2 mmHg), Pramsohler et al. 2017 (hypoxia: systolic: ± 0 mmHg, diastolic: ± 0 mmHg), Wang et al. 2007 (hypoxia: systolic: + 3 mmHg, diastolic: + 2 mmHg), and Wiesner et al. 2009 (systolic: − 2 mmHg, diastolic: − 3 mmHg). Notably, endurance training does not always change blood pressure neither in normotensive nor in hypertensive individuals (Cornelissen and Smart 2013).

Hypoxic exercise training has been shown to decrease arterial stiffness (Nishiwaki et al. 2010), metabolic risk factors like body fat (Haufe et al. 2008; Wiesner et al. 2009) and insulin resistance (Haufe et al. 2008; Mackenzie et al. 2011; Morishima et al. 2013), which all have been implicated in the pathogenesis of hypertension. As an acute effect, endurance exercise as wells as hypoxia result in vasodilation (Nishiwaki et al. 2010) and decreased blood pressure (Somers et al. 1991; Parati et al. 2015). These effects are induced, amongst others, by increased HIF-1α protein expression, triggered by increased oxygen consumption or lowered tissue oxygen tension (Ameln et al. 2005). HIF-1α elicits VEGF activation (Ameln et al. 2005), which acutely affects blood pressure control via nitric oxide synthase expression and nitric oxide activity (Blitzer et al. 1996; Facemire et al. 2009). The activation of the HIF pathway appears to be stronger with endurance exercise performed under hypoxic conditions (Vogt et al. 2001). Therefore, combining these two stimuli might be a reasonable approach to attain synergistic benefits on acute and chronic blood pressure control (Casey et al. 2010; Beck et al. 2013; Pedralli et al. 2020).

Controversial to these positive effects, deleterious reactions may also occur during acute hypoxic exposure (Manukhina et al. 2006). Intermittent episodes of breathing hypoxic air have been used as a model for sleep apnea and caused hypertension in animal models (Fu et al. 2007; Manukhina et al. 2006). Stimulation of the peripheral chemoreceptors, which leads to an increase in sympathetic tone and a decrease in vagal activity, can cause an acute increase in blood pressure (Fu et al. 2007). However, these adverse effects seem to occur during severe and/or long lasting hypoxic exposure (Manukhina et al. 2006), not comparable to short-term exposure as applied in our study.

We did not observe improvements in cardiorespiratory fitness (VO_2_peak) after 8 weeks, which might indicate that our training program is unlikely to change other health-related outcomes. We chose this protocol as it reflects guideline recommendations (ACSM 1993) and is considered to positively affect cardiorespiratory fitness and blood pressure even with small sample sizes (Badenhop et al. 1983). Despite no change in *V*O_2peak_, maximal power increased significantly in both study groups, which indicates a partially successful training protocol with regard to physical fitness.

While the observed systolic office blood pressure reduction in our study (2 mmHg) did not reach statistical significance in either group, the magnitude of pressure reduction is similar to those reported in previous meta-analysis (Cornelissen and Fagard 2005; Cornelissen and Smart 2013). Although the change was small, meta-analyses suggest that a 2 mmHg systolic blood pressure decrease reduces the risk of major cardiovascular disease events by about 4%, stroke by 5%, and heart failure by 6% (Ettehad et al. 2016; Cornelissen and Fagard 2005). Hence, our training program showed potential to exert clinically relevant reductions in office blood pressure; however, normobaric hypoxia did not further improve these responses compared with exercise alone.

As no adverse effects or discomfort occurred during our training program, normobaric hypoxic training at simulated 2700 m altitude (F_I_O_2_ = 0.15%) appears to be feasible to apply in old individuals. A potential benefit of hypoxic training could be the performance of exercise at reduced work rate and thereby less stress on the locomotor system. This was reported by some studies (Haufe et al. 2008; Pramsohler et al. 2017) whereas others did not report such differences. We observed a 15% lower work rate at average for those exercising under normobaric hypoxia, which does not reach statistical significance. Nevertheless this data imply to further studying hypoxic training in larger, but also various target groups as an approach for attaining similar cardiovascular or metabolic benefits after exercise training, while performing at lower intense work rates.

Our study has strengths and limitations. Blood pressure changes were assessed at rest and during exercise in a population in which the risk for hypertension is elevated. Although continuous physical activity would be important for these individuals, this task is often limited by locomotor constriction, which may offer a potential for hypoxic training because of reduced work rate.

As a limitation, the prescription of exercise intensity based on pre-training %*V*O_2_max may have produced different training stimuli across participants, which may have led to a training stimulus not sufficient to induce changes in blood pressure in some participants. We used an intention-to-treat analysis with baseline observation carried forward for missing data as a conservative statistical method, as it avoids possible bias associated with loss of patients in a controlled trial. However, since this approach has also potential weaknesses, we conducted a per-protocol analysis (only cases with pre- and post-intervention data) in addition to our a priori defined analysis.

In conclusion, 8 weeks of hypoxic exercise in old women and men is a safe and feasible training method. However, it is not superior over normoxic training at the same relative exercise intensity. It needs to be further examined if longer durations of hypoxic episodes or higher training intensities have more beneficial effects.

## Data Availability

The datasets generated during and/or analyzed during the current study are available from the corresponding author on reasonable request.

## Abbreviations

ANCOVA: Analysis of covariance
BMI: Body mass index
F_I_O_2_: Fraction of inspired oxygen
HIF-1α: Hypoxia inducible factor 1α
HOMA-IR: Homeostasis model assessment insulin resistance index
RPE: Rating of perceived exertion
SaO_2_: Arterial oxygen saturation
SD: Standard deviation
VEGF: Vascular endothelial growth factor
VO_2peak_: Peak oxygen consumption
